# Rules of Individual Hygiene*Summary of Yale lectures on Hygiene, 1906-7.

**Published:** 1909-07

**Authors:** Irving Fisher


### Abstracts and Selections.

### Rules of Individual Hygiene.*

BY IRVING FISHER.

Preventable death and sickness are the joint results of an inefficient public health service and a defective personal hygiene. Every individeual has two barriers against sickness and death, the first that afforded by the State, in quarantine, cleanliness of streets, purity of water and the like, and the second, individual resistance. The latter barrier—a high potential resistance—is the most effective protection we have in warding off sickness and death, even after dangerous exposure. Resistance is the product of individual hygiene. Individual hygiene comprises a few simple rules religiously adhered to. The essentials of success are knowledge, self-control, enthusiasm.

The essential rules cover plenty of fresh air, both for the lungs and skin, proper bathing, exercising, resting, sleeping, thinking, feeling and willing. Almost all changes of habits require a period of physiological adjustment, and, therefore, should be made gradually. The foregoing lead to the following specific rules:

### AIR.

Keep outdoors as much as possible.

Breathe through the nose, not through the mouth.

When indoors have the air as fresh as possible—

(a) By having aired the room before occupancy.

(b) By having it continuously ventilated while occupied.

(In winter the ventilation is best secured by a window-board deflecting the entering cold air upward.)

---

*Summary of Yale lectures on Hygiene, 1906-7.

Not only purity, but coolness, dryness and motion of the air, if not very extreme, are advantageous. Air in heated houses in winter is usually too dry, and may be humidified with advantage.

Clothing should be sufficient to keep one warm. The minimum that will secure this result is the best. Porosity is very important, not only in underclothes, but in all clothes. The more porous the clothes, the more the skin is educated to perform its functions with increasingly less need for protection. Take an air bath as often and as long as possible.

### WATER.

Take a daily water bath, not only for cleanliness, but for skin gymnastics. A cold bath is better for this purpose than a hot bath. A short hot followed by a short cold bath is still better. In fatigue, a very hot bath lasting only half a minute is good.

A neutral bath, beginning at 97 or 98 degrees, dropping not more than 5 degrees, and continued 15 minutes or more, is an excellent means of resting the nerves.

Be sure that the water you drink is free from dangerous germs and impurities. "Soft" water is better than "hard" water. Ice water should be avoided unless sipped and warmed in the mouth. Ice may contain spores of germs even when germs themselves are killed by cold.

Cool water drinking, including especially a glass half an hour before breakfast and on retiring, is a remedy for constipation.

The judicious use of enemas is advantageous where there is auto-intoxication—that is, absorption of poisons through the colon. They are especially needed when one is not feeling well from almost any cause, as a cold. A warm enema is likely to have an after effect, the inability to defecate without its use. For this reason cool enemas—temperature of 80 degrees down to 75 degrees—are best. The best way, however, of regulating the bowels is by exercise and diet.

### FOOD.

Teeth should be brushed thoroughly several times a day, and floss silk used between the teeth. Persistence in keeping the mouth clean is not only good for the teeth, but for the stomach.

Masticate all food up to the point of involuntary swallowing, with the attention on the taste, not on the mastication. Food should simply be chewed and relished with no thought of swallowing. There should be no more effort to prevent than to force swallowing. It will be found that if you attend only to the agreeable task of extracting the flavors of your food, nature will take care of the swallowing and this will become, like breathing, involuntary. The more you rely on instinct, the more normal, stronger and surer the instinct becomes. The instinct by which most people eat is perverted through the "hurry habit" and the use of abnormal foods. Thorough mastication takes time, and, therefore, one must not feel hurried at meals if the best results are to be secured.

Sip liquids, except water, and mix with saliva as though they were solids.

The stopping point for eating should be at the earliest moment when one is really satisfied. Normalized instinct is the best guide here, provided one eats slowly.

The frequency of meals and time to take them should be so adjusted that no meal is taken before a previous meal is well out of the way, in order that the stomach may have had time to rest and prepare new juices. Normal appetite is a good guide in this respect. One's best sleep is on an empty stomach. Food puts one to sleep by diverting blood from the head, but disturbs sleep later. Water, however, or even fruit, may be taken before retiring without injury.

An exclusive diet is usually unsafe. Even foods which are not ideally the best are probably needed when no better are available, or when the appetite especially calls for them.

The amount of protein required is much less than that ordinarily consumed. Through thorough mastication the amount of protein is automatically reduced to its proper level.

The sudden or artificial reduction in protein to the ideal standard is apt to produce temporarily a "sour stomach," unless fats be used abundantly.

To balance each meal is of the utmost importance. When one can trust the appetite, it is an almost infallible method of balancing, but some knowledge of foods will help. The location on the "food map"* and the knowledge of the ideal position for each individual in the "normal rectangle" will help, especially at first. The aim, however, should always be—and this can not be too often repeated—to educate the appetite to the point of deciding all these questions automatically.

The character of the fees is greatly improved if the diet is proper in respect to protein, and is properly eaten with respect to mastication; otherwise there is almost always absorption of poisons through the colon. Thorough mastication, moderation in amount—especially of proteins—are the best disinfectants. The use of sour milk has an advantage mentioned by Metchnikoff by reducing the bacteria in the colon. There is, therefore, great hygienic value in sour milk, buttermilk, lactic acid koumiss (not the same as yeast-made koumiss), kefir, yoghourd, etc.

### EXERCISE AND REST.

The hygienic life should have a proper balance between rest and exercise of various kinds, physical and mental. Generally every muscle in the body should be exercised daily.

Muscular exercise should hold the attention, and call into play will power. Exercise should be enjoyed as play, not endured as work.

The most beneficial exercises are those which stimulate the action of the heart and lungs, such as rapid walking, running, hill climbing and swimming.

The exercise of the abdominal muscles is the most important in order to give tone to those muscles and thus aid the portal circulaation. For the same reason erect posture, not only in standing but in sitting, is important. Support the hollow of the back by a cushion or otherwise. A rocker or a tilted chair is restful to the portal circulation if the lower back is properly supported. Breathing exercises, both by suction and otherwise, for pumping the portal circulation free of stagnated blood, are very helpful.

Exercise should always be limited by fatigue, which brings with it fatigue poisons. This is nature's signal when to rest. If one's use of diet and air are proper, the fatigue point will be much further off than otherwise.

One should learn to relax when not in activity. The habit produces rest, even between exertions very close together, and enables one to continue to repeat those exertions for a much longer time than otherwise. The habit of lying down when tired is a good one.

The same principles apply to mental rest. Avoid worry, anger, fear, excitement, hate, jealousy, grief and all depressing or abnormal mental states. This is to be done not so much by repressing these feelings as by dropping or ignoring them—that is by diverting and controlling the attention. The secret of mental hygiene lies in the direction of attention. One's mental attitude, from a hygienic standpoint, ought to be optimistic and serene, and this attitude should be striven for not only in order to produce health, but as an end in itself, for which in fact even health is properly sought. In addition, the individual should, of course, avoid infection, poisons and other dangers.

Occasional physical examinations by a competent medical examiner is advisable. In case of illness, competent medical treatment should be sought.

Finally the duty of the individual does not end with personal hygiene. He should take part in the movements to secure better public hygiene in city, State and nation. He has a selfish as well as an altruistic motive to do this. His air, water and food depend on health legislation and administration.

All the foregoing rules are important. The results which may be obtained by following them depend largely on the thoroughness with which they are followed. This is true especially of fresh air and mastication. If all the rules are followed and followed thoroughly, including the one most commonly neglected, namely, keeping within the fatigue limit, the average man may reasonably expect if not to equal the record of Cornaro at least to double his length of life, his activity per day, his satisfactions and his usefulness. The laws of "humaniculture" can be depended upon as much as those of agriculture, horticulture or stock raising.—*American Health.*

---

NOT all soft swellings of the axilla are to be incised. Aneurism of the axillary artery has been mistaken for abscess.—*American Journal of Surgery.*

